# Metabolomics in Diabetic Retinopathy: From Potential Biomarkers to Molecular Basis of Oxidative Stress

**DOI:** 10.3390/cells11193005

**Published:** 2022-09-26

**Authors:** Qizhi Jian, Yingjie Wu, Fang Zhang

**Affiliations:** 1 National Clinical Research Center for Eye Diseases, Shanghai General Hospital, Shanghai Jiao Tong University School of Medicine, Shanghai 200080, China; 2 Shanghai Key Laboratory of Ocular Fundus Diseases, Shanghai 200080, China; 3 Shanghai Engineering Center for Visual Science and Photomedicine, Shanghai 200080, China; 4 Shanghai Engineering Center for Precise Diagnosis and Treatment of Eye Diseases, Shanghai 200080, China; 5 Institute for Genome Engineered Animal Models of Human Diseases, National Center of Genetically Engineered Animal Models for International Research, Liaoning Provence Key Laboratory of Genome Engineered Animal Models, Dalian Medical University, Dalian 116000, China; 6 Shandong Provincial Hospital, School of Laboratory Animal & Shandong Laboratory Animal Center, Science and Technology Innovation Center, Shandong First Medical University & Shandong Academy of Medical Sciences, Jinan 250021, China; 7 Department of Molecular Pathobiology, New York University College of Dentistry, New York, NY 10010, USA

**Keywords:** diabetic retinopathy, metabolomics, biomarkers, metabolic pathway, molecular targets

## Abstract

Diabetic retinopathy (DR), the leading cause of blindness in working-age adults, is one of the most common complications of diabetes mellitus (DM) featured by metabolic disorders. With the global prevalence of diabetes, the incidence of DR is expected to increase. Prompt detection and the targeting of anti-oxidative stress intervention could effectively reduce visual impairment caused by DR. However, the diagnosis and treatment of DR is often delayed due to the absence of obvious signs of retina imaging. Research progress supports that metabolomics is a powerful tool to discover potential diagnostic biomarkers and therapeutic targets for the causes of oxidative stress through profiling metabolites in diseases, which provides great opportunities for DR with metabolic heterogeneity. Thus, this review summarizes the latest advances in metabolomics in DR, as well as potential diagnostic biomarkers, and predicts molecular targets through the integration of genome-wide association studies (GWAS) with metabolomics. Metabolomics provides potential biomarkers, molecular targets and therapeutic strategies for controlling the progress of DR, especially the interventions at early stages and precise treatments based on individual patient variations.

## 1. Introduction

Diabetic retinopathy (DR) is a major complication of diabetes mellitus (DM), and one of the leading causes of vision impairment and blindness in working-age adults globally [1,2,3,4]. In 2030, the number of adults worldwide with DR is estimated to be 129.84 million, and the number is projected to increase to 160.50 million in 2045 [5]. The economic burden increased accordingly.

Although important advances have been made in the diagnosis and treatment of DR in the past few decades, more effective diagnostic markers and therapeutic strategies are still lacking. Hemoglobin A1c (HbA1c) for monitoring the levels of glucose is the validated systemic biomarker of DR [6], while the differences in the level of HbA1c explained only 6.6% of the alteration in the risk of DR for the entire study cohort in a diabetes control and complications trial [7,8]. The worsening of DR, including the early-phase non-proliferative diabetic retinopathy (NPDR) and advanced-phase proliferative diabetic retinopathy (PDR), is associated with the initiation of effective treatment of glycaemia in patients with diabetes. Moreover, there are few measures available for early intervention in DR beyond regulating hyperglycemia and hypertension, preventing dyslipidemia, and cessation of tobacco smoking [9]. All the treatments including anti-vascular endothelial growth factor (anti-VEGF) therapy and laser photocoagulation are focused on the PDR and carry sight-threatening effects [9,10,11]. Thus, there is still an urgent need to identify novel biomarkers and effective therapeutic strategies to screen and treat the progression of DR.

Research progress supports that oxidative stress, caused by the disruption of redox balance, is closely related to metabolic dysregulation in the retina and is a key contributor to the pathogenesis of DR [12,13]. Metabolism-based regulation of oxidative stress would be a potential target for developing promising treatments for DR.

Metabolomics enables the detailed characterization of metabolic phenotypes and metabolic derangements that underlie diseases. It could afford the discovery of biomarkers and new therapeutic targets that may be used to either diagnose disease or monitor the activity of therapeutics [14]. The application of metabolomics in DR has systematically reflected abnormal metabolic changes by comparing the small molecule composition of various ocular and blood samples from DR patients, mammals, tissues, and cells [15]. In this manuscript, we aim to review the latest advances in metabolomics of DR and summarize potential biomarkers and molecular targets for DR by integrating metabolomics with genome-wide association studies (GWAS).

## 2. Overview of Metabolomics of DR

**Figure 1:**
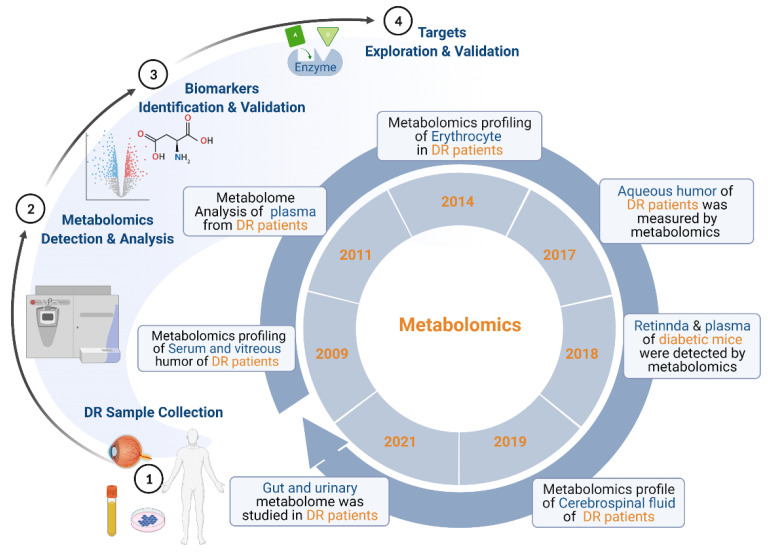
The applications of metabolomics in diabetic retinopathy. Since 2009, metabolomics studies of DR using various biological samples have become widely available. After sample collection and metabolomics detection and analysis, differential metabolites are obtained, which can be applied to identify biomarkers and explore metabolic targets.

In recent years, an increasing number of studies have applied metabolomics in DR. We retrieved relevant articles by searching PubMed before 28 February 2022, with the following search method: (“metabolomics” or “lipidomics” or “metabonomics” or “metabolome” or “metabolic profiling”) AND “diabetic retinopathy”. Thirty-nine original articles were finally determined and the details of these articles including species, samples, platforms, and differential metabolites are summarized in Figure 1 and Table 1.

According to the search results, metabolomics studies of DR using various biological samples have become widely available since 2009 (Figure 1). In 2009, Abhary et al. performed metabolomics profiling of serum from patients with DR and found that the serum levels of L-arginine, asymmetric dimethylarginine (ADMA), and symmetric dimethylarginine (SDMA) were increased in DR patients compared to diabetic patients without DR [26]. In the same year, Young et al. used metabolomic analysis of human vitreous humor to differentiate ocular inflammatory diseases including proliferative vitreoretinopathy (PVR) and proliferative diabetic retinopathy, and showed that PVR and PDR could be separated by the metabolomic analysis of vitreous humor [39]. In 2011, Li et al. analyzed the metabolome of plasma from patients with DR and identified pyruvic acids, l-aspartic acid, β-hydroxybutyric acid, methylmaleic acid, citric acid, glucose, stearic acid, trans-oleic acid, linoleic acid, and arachidonic acid as differential metabolites [16]. Koehrer et al. identified the metabolic profiles of erythrocytes in DR patients and found that the levels of docosahexaenoic acid, arachidonic acid, and PUFAs in red blood cells were decreased in 2014 [32]. The metabolic profile of aqueous humor from patients with DR was measured by Kunikata et al. in 2017 [37]. They identified cysteine persulfides, oxidized glutathione trisulfide (GSSSG) and cystine were changed in aqueous humor. The next year, Sas et al. examined the lipidome in plasma and retinal tissues using a mouse model of type 2 diabetes with microvascular complications and found alterations of 15 lipids in both plasma and retina tissues [52]. Lin et al. investigated the metabolomic profile of type 2 diabetes in cerebrospinal fluid and identified that alanine, histidine, leucine, pyruvate, tyrosine, and valine showed the potential of biomarkers for DR in 2019 [47]. In 2021, Ye and Zhou respectively collected stool samples from DR patients to analyze the alterations of gut metabolomes linking DR to the gut metabolome—gut-retina-axis [33,34]. Meanwhile, Quek et al. analyzed the urinary metabolic profiles of DR patients and found the alterations of citrate, ethanolamine, formate, and hypoxanthine in urine [50].

From the perspective of species, 35 studies recruited patients with DR for metabolomic analysis, and the other four studies selected diabetic animal models including mice, rats and zebrafish. There is a wide selection of biofluids for metabolomic analysis in human studies, including circulating blood (plasma and serum), eye fluids (vitreous humor and aqueous humor), and other samples (retinal tissues, stools, urines, red blood cells, and cerebrospinal fluids). Different types of samples have their own characteristics and advantages. Circulating blood, due to its easier availability and lower invasiveness, is the most commonly used sample and can provide a global metabolomic picture [55]. Both serum and plasma can be obtained from blood, and the main difference between them is the presence or absence of clotting factors [56]. In terms of impact on metabolite detection, plasma appears to have better reproducibility, with serum having higher concentrations [57]. Eye fluids including vitreous humor and aqueous humor can directly reflect intraocular metabolic variations. However, the vitreous humor, a highly aqueous eye fluid interfacing with the retina, can only be obtained from subjects with PDR during surgery such as a vitrectomy, which results in the absence of vitreous samples of NPDR. Tears can be obtained non-invasively and can also reflect the conditions of the oculi posterior segment [6]. However, to our knowledge, tear metabolomics have not been applied to the study of DR. In addition, stool samples can reflect alterations of fecal metabolome and gut microbiota composition, linking DR to the gut metabolome and microbiota—gut-retina-axis [58].

From the perspective of the metabolomics analysis platform, there are two main tools: nuclear magnetic resonance (NMR) spectroscopy and mass spectrometry (MS). Thirty-three studies used MS for metabolite analysis and five studies used NMR. NMR spectroscopy can be applied to biological samples in various states including liquid, solid, and gaseous samples [59]. The proton NMR method is the most widely applied NMR technique [60]. A significant advantage of NMR is the small number of samples required [15]. MS is often used in tandem with liquid chromatography (LC) or gas chromatography (GC), which are techniques applied to separate metabolites. In particular, LC-MS has been widely used in recent years. MS has far better sensitivity than NMR, allowing it to measure a wider spectrum of metabolites [15]. Overall, the use of NMR and MS has greatly facilitated the development of metabolomics.

## 3. Potential Metabolomics Biomarkers of DR

Metabolomics has been utilized extensively for the identification of single metabolites and their use as biomarkers [61]. In DR research, 14 studies applied explicit statistical methods to identify new metabolomics biomarkers and evaluate the performance for disease diagnosis of biomarker models as listed in Table 2. These studies primarily covered the human serum, plasma, vitreous humor, aqueous humor, cerebrospinal fluid, and stool. Human plasma is the most widely used sample for identifying biomarkers of DR, and various metabolites in human plasma have been reported to have biomarker potentials. The biomarker potential of cytidine in plasma was reported in two studies [17,22]. The vitreous humor is another biological sample that has been extensively studied to explore novel biomarkers for DR. Haines and Wang confirmed the biomarker potential of pyruvate in the vitreous humor of PDR patients, respectively [38,43]. Predicted biomarkers and their diagnostic performance are detailed below.

### 3.1. Potential Biomarkers in Human Serum

There are two studies that documented potential biomarkers of DR in human serum [27,30]. Xuan et al. used multiplatform-based metabolomics to generate the metabolic profile of serum samples from 689 subjects with DR and 216 subjects with diabetes without DR [27]. The biomarker panel containing 12-hydroxyeicosatetraenoic acid (12-HETE) and 2-piperidone exhibited good performance for DR diagnosis. The AUC, sensitivity, and specificity of this panel were 0.946, 0.894, and 0.919, respectively, suggesting a potential value as a biomarker for differentiating DR from diabetes. Notably, the biomarker panel also exhibited good performance in differentiating NPDR from diabetes (AUC = 0.958, sensitivity = 0.929, specificity = 0.901). Zuo et al. performed a widely targeted metabolomics based on ultra-performance liquid chromatography-electrospray ionization-tandem mass spectrometry (UPLC-ESI-MS/MS) in the serum samples from 69 subjects with DR and 69 subjects with diabetes without DR [30]. A biomarker model called multidimensional network biomarkers consisting of linoleic acid, nicotinuric acid, ornithine, and phenylacetylglutamine was established. The AUC, sensitivity, and specificity of the MDNBs were 0.92, 0.96, and 0.78, respectively.

### 3.2. Potential Biomarkers in Human Plasma

Eight studies have reported potential novel biomarkers for DR in human plasma [17,18,19,20,22,24,47,48]. Xia et al. investigated the relationship between pyrimidine metabolites and DR, and identified cytidine as a potential biomarker (AUC = 0.849, sensitivity = 0.737, specificity = 0.919) [17]. Similarly, Xia et al. investigated the relationship between purine metabolites and DR, and identified adenosine as a potential biomarker (AUC = 0.913, sensitivity = 0.947, specificity = 1) [18]. Chen et al. performed metabolomics using GC-MS and found that 1,5-gluconolactone, 2-deoxyribonic acid, gluconic acid, and urea exhibited the potential of a biomarker (AUC = 0.71, 0.68, 0.72, 0.69, respectively) [19]. Rhee et al. recruited 183 patients with type 2 diabetes (52 PDR, 72 NPDR, and 59 NDR) and analyzed their plasma metabolic profiles using ultra-performance liquid chromatography–quadrupole/time-of-flight mass spectrometry (UPLC–Q–TOF–MS) and gas chromatography (GC)–TOF–MS [20]. Their results show that glutamine and glutamic acid were the most differential metabolites and their ratio showed a potential diagnostic value for DR (AUC = 0.742). To identify novel metabolite markers for PDR, Zhu et al. performed metabolomics based on LC-MS in 21 subjects with PDR and 21 subjects with a duration of diabetes of ≥10 years but without DR, and found fumaric acid, uridine, acetic acid, and cytidine to have biomarker potentials (AUC = 0.96, 0.95, 1.0, 0.95, respectively) [22]. Notably, the biomarker potential of cytidine was again demonstrated, which is consistent with the previous study by Xia et al. [17]. Sun et al. recruited 21 patients with PDR, 21 patients with NPDR and 32 patients with type 2 diabetes without DR, and used ultrahigh-performance liquid Q-Exactive mass spectrometry (UPLC-QE-MS) to analyze plasma’s metabolic profile [24]. They established a formula based on the plasma concentration of pseudouridine to calculate the DR risk score: risk score = −0.23 × Ln (pseudouridine) + 1.88. The AUC of the risk score for DR was 0.80, with 97.6% sensitivity and 53.1% specificity. Another formula based on the levels of pseudouridine, N-acetyltryptophan, leucylleucine, and glutamate, was established to calculate the PDR risk score: risk score = 0.23 × Ln(pseudouridine) + 0.16 × Ln(N-acetyltryptophan)-0.065 × Ln(leucylleucine) + 0.11 × Ln(glutamate) − 3.63. The AUC of the risk score for PDR was 0.82, with 76.2% sensitivity and 77.4% specificity. Curovic et al. performed metabolomics and lipidomics analyses to generate the metabolic profile related to DR in 648 individuals with type 1 diabetes [48]. Cox proportional hazard model analysis showed that higher 3,4-dihydroxybutyric acid (3,4-DHBA) was an independent risk marker for DR progression (HR 1.55, 95% CI 1.12–2.15, *p* = 0.033).

### 3.3. Potential Biomarkers in Human Vitreous Humor

A total of four studies have investigated potential biomarkers for DR in human vitreous humor [38,40,43,46]. Barba et al. acquired 1H-NMR spectra from vitreous samples of 22 subjects with type 1 diabetes with PDR and 22 non-diabetic subjects, and obtained a model consisting of galactitol and ascorbic acid (AA) that can distinguish PDR and control with 86% sensitivity and 81% specificity [40]. Haines et al. analyzed the vitreous humor of nine patients with PDR and eight non-diabetic patients using UPLC-MS [43]. They performed biomarker analysis using ROC curves, showing that xanthine, proline, citrulline, and pyruvate were the strongest potential predictors of DR (AUC = 1.0, 0.986, 0.972, 0.944 respectively). Wang et al. used gas chromatography coupled with time-of-flight mass spectrometry (GC-TOFMS) to identify potential DR biomarkers in vitreous humor from 28 subjects with type 2 diabetes with PDR and 22 non-diabetic subjects [38]. They found a biomarker panel consisting of pyroglutamic acid and pyruvic acid (AUC = 0.951, sensitivity = 0.955, specificity = 0.857). Zhao et al. performed targeted lipidomics to evaluate oxylipin levels in the vitreous humor using ultra-high-performance liquid-chromatography-multiple reaction monitoring-mass spectrometry/mass spectrometry (UHPLC-MRM-MS/MS) [46]. Vitreous samples were collected from 41 subjects with PDR and 22 non-diabetic subjects. Oxylipins are oxidation products of polyunsaturated fatty acids (PUFAs). According to their results, seven oxylipins were considered as potential biomarkers: docosatetraenoic acid (DTA), eicosapentaenoic acid (EPA), docosahexaenoic acid (DHA), arachidonic acid (ARA), ±9(10)-dihydroxy-octadecenoic acid (±9(10)-DiHOME), ±19.20-epoxy-docosapentaenoic acid (±19,20-EpDPE), and ±12(13)- epoxy-octadecenoic acid (±12(13)-EpOME) (AUC = 0.96, 0.803, 0.871, 0.942, 0.805, 0.819, 0.828, respectively).

### 3.4. Potential Biomarkers in Other Human Samples

DR biomarkers have been predicted in the aqueous humor, cerebrospinal fluid, and feces of humans in three metabolic studies [33,38,47]. Wang et al. also identified potential DR biomarkers in aqueous humor using GC-TOFMS [38]. They recruited 23 subjects with type 2 diabetes with PDR and 25 non-diabetic subjects with cataract, and found a biomarker model consisting of D-2,3-dihydroxypropanoic acid, isocitric acid, fructose 6-phosphate, and L-lactic acid. The AUC of the model was 0.965 with 88% sensitivity and 95.7% specificity. Lin et al. were the first to investigate the metabolomic profile of type 2 diabetes in cerebrospinal fluid [47]. Their study cohort included 19 patients with DR and 14 patients with type 2 diabetes without diabetic microangiopathy. They constructed a multi-marker panel established by alanine, histidine, leucine, pyruvate, tyrosine, and valine showing a high relevance to the occurrence of DR with 0.858 AUC. This multi-marker panel was also validated in plasma with 0.836 AUC. Ye et al. performed 16S rRNA gene sequencing and UPLC-MS-based untargeted metabolomics of fecal samples to investigate the gut metabolome and microbiome [33]. They collected fecal samples from 45 subjects with PDR and 90 subjects with type 2 diabetes without DR. They established a fecal metabolite-based classifier to differentiate PDR and NDR with AUCs of 0.960 and 0.943 in train and test sets. The top 5 differential metabolites in the classifier are alantolactone, desogestrel, adenine, D-erythro-sphinganine, and corosolic acid.

## 4. Metabolic Pathways Associated with DR

To gain an in-depth understanding of the mechanism underlying metabolic disorders in DR, we counted the differential metabolic pathways reported in plasma and vitreous from DR patients. Purine metabolism, pyrimidine metabolism, arginine and proline metabolism, and glutamate metabolism are the most frequently reported differential pathways in DR metabolomics studies. Details about differential metabolic pathways are summarized in Table 3.

### 4.1. Pyrimidine Metabolism

Pyrimidine metabolism disorder has been reported in the blood of patients with DR [17,22,23]. Derivatives of pyrimidine exhibit highly potential biological activity as anti-diabetic agents [62,63]. In previous studies [17,22], changed levels of cytidine, a pyrimidine molecule, was observed in patients with DR. Cytidine is the precursor of cytidine triphosphate (CTP), which affects phosphatidylcholine (PC) and phosphatidylethanolamine (PE) biosynthetic pathways. Previous studies reported that phospholipid metabolism is associated with diabetic nephropathy, and that the level of phospholipids decreased with the development of diabetic nephropathy [64]. The mechanism of pyrimidine metabolism in the onset and development of DR still needs further identification and exploration.

### 4.2. Glutamate Metabolism and Branched-Chain Amino Acid (BCAA) Metabolism

Glutamate metabolism is another affected abnormal metabolic pathway in DR [38]. Glutamate is not only a key signal in the amplification of insulin secretion [65], but is also the major excitatory neurotransmitter in the central nervous system and retina [66,67]. Several studies found increased glutamate and decreased glutamine levels in the vitreous humor of patients with PDR and in diabetic rat retina [68,69,70]. The increased level of glutamate in the retina will cause neurotoxic effects and the activation of ionotropic glutamate receptors in excess, mainly the N-methyl-d-aspartame receptor (NMDAR), resulting in uncontrolled intracellular calcium responses and cell death [71,72,73]. Meanwhile, the levels of leucine, isoleucine, and valine in BCAA metabolism were increased in the serum of DR patients and in the diabetic rat retina [27,74], which are considered to be correlated with the neurotoxic effects of glutamate, which plays an important role in DR neurodegeneration [74]. Therefore, more attention to the abnormal glutamate metabolism and BCAA metabolism may contribute to understanding the pathogenesis of DR.

### 4.3. Pantothenate and CoA Biosynthesis

Notably, pantothenate and CoA biosynthesis were also altered in both the plasma and vitreous humor of patients with DR [49]. Wang et al. discovered a descending trend of pantothenate in the plasma of PDR patients and an ascending trend of pantothenate in the vitreous [49]. Ma et al. found that the levels of pantothenate and CoA biosynthesis were significantly down-regulated in the urine of patients with diabetic kidney disease [75], which was consistent with Wang’s result in plasma. This phenomenon can probably be explained by a lower pantothenate conversion due to impaired renal tubular reabsorption of vitamins in patients with diabetes complications [76]. A possible explanation for up-regulated levels of pantothenate and CoA biosynthesis in vitreous humor is the mechanism of protecting retinal cells from damage [49]. Endothelial cells were protected from oxidative stress by supplementation with pantothenate [77,78]. Alteration in pantothenate and CoA modulate mitochondrial energy metabolism [79], which is most likely linked to the onset and progression of DR.

### 4.4. Polyol Pathway

Evidence suggests that the polyol pathway can exacerbate oxidative stress to promote the progression of retinopathy [80]. In the hyperglycemic condition, the polyol pathway of glucose metabolism becomes active in human and rat retinal cells [81,82]. In the polyol pathway, glucose is reduced to sorbitol by aldose reductase (AR), and sorbitol is subsequently oxidized to fructose by sorbitol dehydrogenase (SDH). Fructose can be converted to fructose-3-phosphate by phosphorylation, and then fructose-3-phosphate can be transferred to 3-deoxyglucosone, both of which can be involved in the formation of advanced glycation end products (AGEs) [80]. Reactive oxygen species (ROS) induced by AGEs participate in the oxidative stress process of DR, leading to the impairment of retinal vessels [80]. Secondly, AR can convert NADPH to NADP+ and SDH can convert NAD+ to NADH in the polyol pathway. During the reaction, NADPH is consumed in excess, which results in the reduced synthesis of glutathione (GSH) and the weakened capacity against oxidative stress [83]. In summary, the polyol pathway triggered by hyperglycemia can produce AGEs precursors and expose retinal cells to oxidative stress.

## 5. Predictions of Metabolism-Based Molecular Targets in DR

To explore potential metabolic enzymes with regulatory potential in DR, we integrated enzymes in DR-related metabolic pathways with a genome-wide association study (GWAS). GWAS refers to multi-center, large sample, and repeatedly verified association studies between genes and diseases at the whole genome level aiming to identify genotype–phenotype associations [84]. Genetic markers (such as SNP) are typed to comprehensively reveal genes related to the onset and development of diseases. Single nucleotide polymorphism (SNP), the most common heritable variation, refers to the polymorphism of the DNA sequence induced by the alteration of a single nucleotide including the conversion or transversion of a single base and the insertion or deletion of bases [85]. GWAS has been successful in identifying risk variants at genetic loci for many diseases including cancers [86,87,88], diabetes [89], and DR [90].

**Figure 2:**
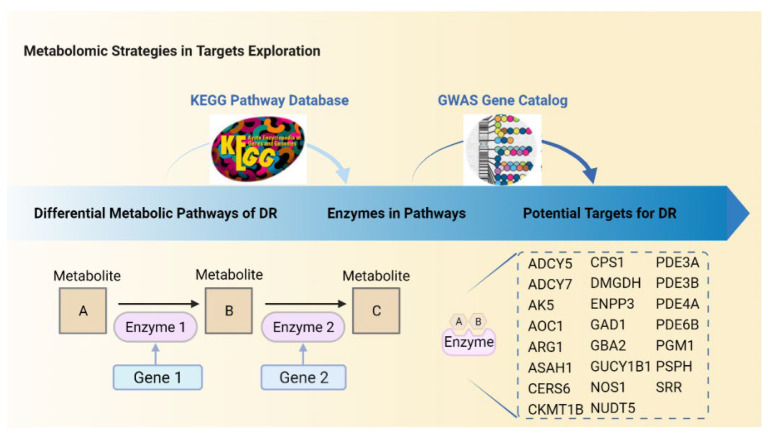
Strategies for exploring potential molecular targets through metabolomics studies. Twenty-three potential regulatory enzymes (genes) were obtained by integrating metabolomics with GWAS. First, the enzyme-related genes in the disordered metabolic pathways were obtained by retrieving metabolic pathways in the KEGG database. Next, SNPs associated with DM or DR were acquired by searching the GWAS Catalog database. Finally, the enzyme-related genes were matched with genes with SNPs.

**Figure 3:**
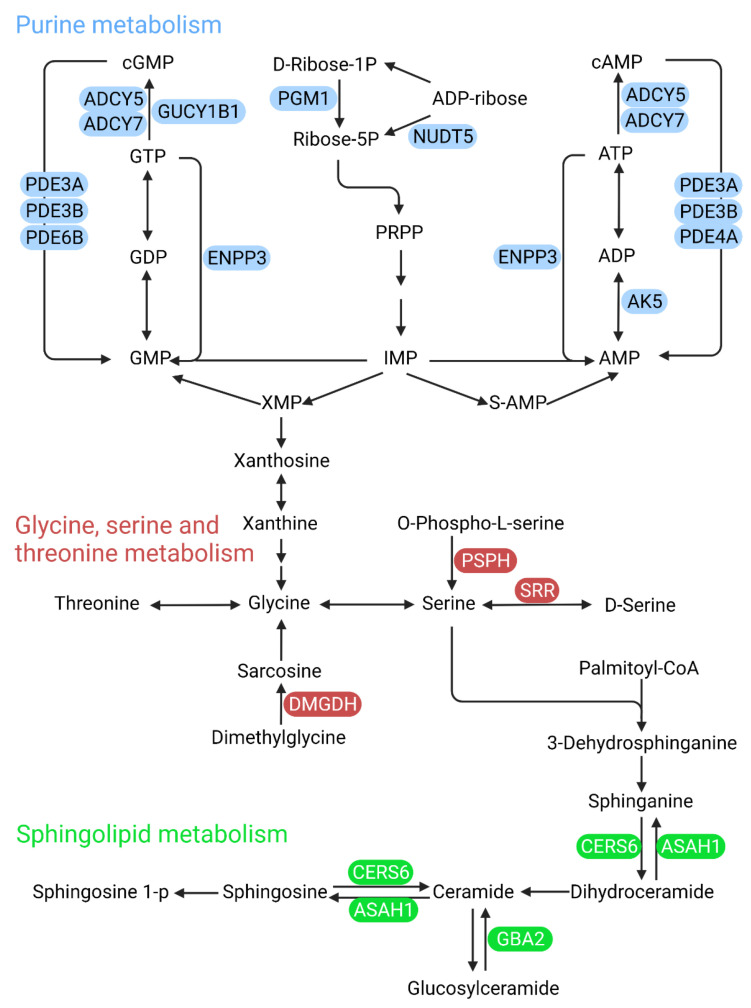
The metabolic network of purine metabolism, glycine, serine and threonine metabolism, and sphingolipid metabolism in DR with potential enzyme targets. Schematic overview of the DR-related metabolic pathways including purine metabolism, glycine, serine and threonine metabolism, and sphingolipid metabolism with related enzymes with SNP depicted in different color schemes. Purine metabolism is depicted in blue, glycine, serine and threonine metabolism in red, and sphingolipid metabolism in green.

**Figure 4:**
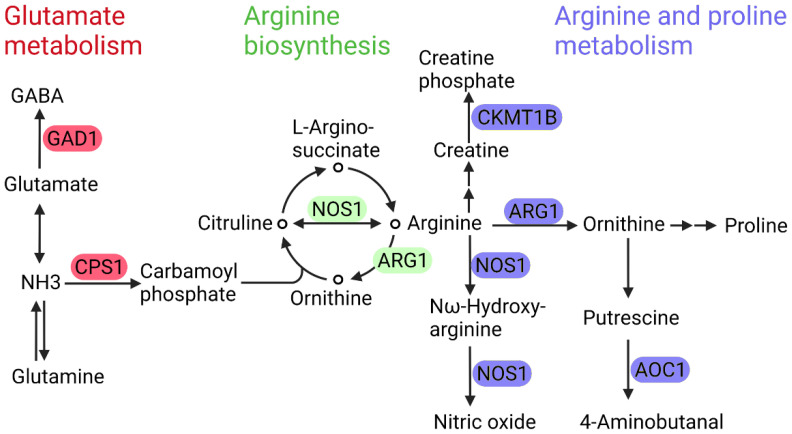
The metabolic network of arginine biosynthesis, arginine and proline metabolism, and glutamate metabolism in DR with potential enzyme targets. Schematic overview of the DR-related metabolic pathways and enzyme genes with SNP. Enzymes involved in arginine biosynthesis, arginine and proline metabolism, and glutamate metabolism are depicted in red, green and blue, respectively.

As shown in Figure 2, a total of 23 enzyme-related genes in 6 DR associated metabolic pathways have SNPs through GWAS database analysis. Among these 23 genes, ADCY5, ADCY7, AK5, ENPP3, GUCY1B1, NUDT5, PDE3A, PDE3B, PDE4A, PDE6B, and PGM1 are involved in purine metabolism, DMGDH, PSPH, and SRR are in glycine, serine and threonine metabolism, and ASAH1, CERS6, and GBA2 are in sphingolipid metabolism (Figure 3). As shown in Figure 4, ARG1, CPS1, and NOS1 are involved in arginine biosynthesis, ARG1, AOC1, CKMT1B, and NOS1 are involved in arginine and proline metabolism, and CPS1 and GAD1 are involved in glutamate metabolism.

All 23 genes encode metabolic enzymes in differential metabolic pathways of DR, and there are SNPs associated with diabetes risk in the 23 genes. These indicated a potential link between these enzymes and the pathogenesis of DR. In purine metabolism, PDE3A, PDE3B, PDE4A and PDE6B are closely associated with retinal degeneration under hypoxic or ischemic conditions [91], and the role of ADCY5, ADCY7, AK5, ENPP3, GUCY1B1, NUDT5, and PGM1 in the onset and development of DR is unknown. In addition, AGR1 and NOS1 are involved in arginine metabolism, which was reported to play an important role in the progression of oxidative stress of DR [92,93].

### 5.1. Arginase 1 and Nitric Oxide Synthase 1

ARG1 encodes arginase 1 catalyzing the hydrolysis of L-arginine to urea and L-ornithine. Ornithine is converted to citrulline, which is converted to arginosuccinate, and finally back to arginine (Figure 4). A recent study showed that high levels of arginase 1 and SNPs (rs2781666 and rs2781665) within the ARG1 are associated with increased type 2 diabetes risk [94]. Elevated concentrations of arginine have been observed in plasma, serum, and the vitreous humor of patients with DR [23,25,26,42]. In addition to the urea cycle, arginine is also involved in the nitric oxide synthesis. Nitric oxide synthase (NOS) catalyzes arginine to citrulline and nitric oxide (NO) [95]. Arginine is the common substrate of nitric oxide synthase and arginase. Under physiological conditions, arginase and NOS compete for the same substrate arginine to produce ornithine and nitric oxide. A study focusing on diabetic cardiomyopathy using H9c2 cells with high glucose treatment found that increased arginase expression results in more arginine flowing to the urea cycle, which reduces the production of NO [96]. In this case, NOS is uncoupled and produces superoxide anions [96]. The superoxide anions react with NO to turn into toxic oxidant peroxynitrite, which is a key indicator of oxidative stress [97]. A previous study has found the increased level of nitrotyrosine, the marker of peroxynitrite, in retinas of the streptozotocin (STZ)-induced diabetic mice [92]. Another study using STZ-induced diabetic mice identified that the inhibition of arginase can reduce nitrotyrosine formation [93]. All of these examples show that ARG1 and NOS1 possess the potential to be regulatory and therapeutic targets for preventing or reversing the oxidative stress of DR.

### 5.2. Phosphodiesterase

Phosphodiesterase (PDE) catalyzes the hydrolysis of cAMP and cGMP, the second messengers that play important roles through multiple intracellular signaling pathways [98,99]. The PDE family consists of 11 members [100]. SNPs within the PDE3A, PDE3B, PDE4A, and PDE6B genes are associated with increased diabetes risk [101,102,103]. A previous study reported that the accumulation of cGMP through inhibiting PDE prevented hypoxia-induced cell death in porcine retinal explants, which reveals the potential for PDE inhibition to reduce retinal degeneration under hypoxic or ischemic conditions [91].

## 6. Conclusions

Over the past dozen years, the metabolomics of DR has experienced great growth. Many works have been undertaken so far in the field of DR for biomarker discovery. For example, 12-HETE and 2-piperidone in serum, cytidine and 3,4-DHBA in plasma, and pyruvate in vitreous were all identified to have great potentials to be biomarkers. Moreover, subtle alterations in biological pathways provide insight into the mechanisms. Twenty-three enzymes in DR associated metabolic pathways show potential as targets. Among these 23 enzymes, AGR1 and NOS1 are closely related to arginine metabolism, which was reported to play an important role in the progression of the oxidative stress of DR. PDE is responsible for the hydrolysis of cyclic nucleotides and is closely associated with retinal degeneration under hypoxic or ischemic conditions.

Promising progress in identifying novel biomarkers has been made, yet there are also many limitations. Firstly, most studies focused on the identification of biomarkers for distinguishing between DR and DM. However, few studies further analyzed biomarkers for different stages of DR, especially the early stage (NPDR), which is very important for early diagnosis and prevention. Secondly, a large number of potential biomarkers found in some studies are difficult to be validated in others. Differences in study design, race and region, and clinical characteristics, as well as small sample sizes in some studies, may lead to this issue. Hence, comprehensive research should be conducted to analyze the numerous discriminant metabolites in different kinds of samples for the purpose of identifying biomarkers with real clinical diagnostic values. In terms of target prediction, some of the predicted molecular targets, such as ADCY5, ADCY7, AK5, ENPP3, GUCY1B1, and NUDT5, have no further experimental evidence to be associated with DR. Among them, ADCY5 and ADCY7 are worthy of further exploration. ADCY5 and ADCY7 encode adenylate cyclase 5 and adenylate cyclase 7, respectively [104]. Several previous GWASs demonstrated that SNPs (for example rs11708067 and rs11717195) in ADCY5 may be associated with type 2 diabetes [101,105,106,107]. Hodson et al. reported that ADCY5 mRNA expression in islets was decreased when subjects have risk alleles at rs11708067 [108]. They showed that ADCY5 is essential to couple glucose to insulin secretion by converting glucose signals into cAMP production. Predicted molecular targets provide broader exploration space for DR research.

Metabolomics is demonstrating its power, from biomarker discovery to understanding the mechanisms that underlie DR. This has also been made possible as metabolomics has become more widely integrated with other omics, such as GWAS. The application of metabolomics in DR might also be expanded for judging and monitoring the precise treatment.

## Figures and Tables

**Table 1:** Summary of published studies on metabolomics of diabetic retinopathy.

Species	Samples	Subjects	Platforms	Differential Metabolites	Study
**Human**	Plasma	64 DR 25 controls	GC–MS	Arachidonic acid, citric acid, glucose, linoleic acid, l-aspartic acid, methymaleic acid, pyruvic acids, stearic acid, trans-oleic acid, β-hydroxybutyric acid	Li et al. (2011) [16]
		38 DR 37 controls	HPLC–MS	↑: cytosine, cytidine, thynidine	Xia et al. (2011) [17]
		39 DR 35 NDR	UPLC-MS	↑: adenosine, inosine, uric acid, xanthine	Xia et al. (2014) [18]
		80 DR 80 controls	GC-MS	↑: erythritol, gluconic acid, lactose/cellobiose, mannose, maltose/trehalose, ribose, urea, 1,5-gluconolactone, 2-deoxyribonic acid, 3,4-dihydroxybutyric acid ↓: 1,5-anhydroglucitol	Chen et al. (2016) [19]
		52 PDR 72 NPDR 59 NDR	UPLC-MS, GC-MS	7 amino acids (asparagine, aspartic acid, glutamic acid, glutamine, glycine, methionine, pyroglutamic acid), 6 organic compounds (citric acid, lactic acid, phosphoric acid, succinic acid, urea, uric acid), 7 carbohydrates (fructose, glucose, myo-inositol, 1,5-anhydroglucitol, 3 saccharides), 11 LysoPCs	Rhee et al. (2018) [20]
		28 NPDR 22 NDR	LC-MS	PGF2α	Peng et al. (2018) [21]
		21 PDR 21 NDR	UPLC-MS	63 metabolites (e.g., acetic acid, cytidine sulfite, dihydrouracil, fumaric acid, imidazolone, L-serine, malonic acid, sulfate, uridine, and β-alanine)	Zhu et al. (2019) [22]
		83 DR 90 NDR	LC-MS	126 metabolites (e.g., arginine, acylcarnitine, argininic acid, citrulline, dehydroxycarnitine, glutamic γ-semialdehyde)	Sumarriva et al. (2019) [23]
		21 PDR 21 NPDR 32 NDR	UPLC-MS	Acetylcarnitine, butyryl carnitine, cholic acid, D-glucuronic acid, D-(+)-pantothenic acid, dehydroisoandrosterone sulfate, pantothenic acid, pseudouridine, hypoxanthine, N2,N2-dimethylguanosine, N-acetyltryptophan, leucylleucine, sn-glycero-3-phosphocholine, propionylcarnitine, inosine, urocanic acid, N-fructosyl isoleucine, kynurenic acid, phenylacetylglutamine, glutamine, (−)-riboflavin, 3-methylhistidine,	Sun et al. (2021) [24]
		64 PDR 92 NPDR 159 NDR	LC-MS	↑: arginine, citrulline	Peters et al. (2021) [25]
	Serum	176 DR 329 NDR	LC-MS	↑: asymmetric dimethylarginine (ADMA), L-arginine, symmetric dimethylarginine (SDMA)	Abhary et al. (2009) [26]
		689 DR 216 controls	GC-MS, LC-MS	12-hydroxyeicosatetraenoic acid (12-HETE) and 2-piperidone	Xuan et al. (2020) [27]
		43 DR 44 controls	UHPLC–MS	↑: 13 lipid (sub)classes (Cers, CerG1s, ChEs, DGs, FAs, LPCs, LPEs, LPC-Os, LPE-ps, PCs, PC-Os, PE-ps, SMs)	Xuan et al. (2020) [28]
		51 PDR 123 NPDR 143 NDR	LC–MS	DR vs. NDR: 62 metabolites PDR vs. NDR: 53 metabolites NPDR vs. NDR: 30 metabolites PDR vs. NPDR: 8 metabolites	Yun et al. (2020) [29]
		69 DR 69 NDR	UPLC-MS	↑: nicotinuric acid, o-cresol, ornithine, phenylacetylglutamine, p-cresol ↓: alpha-linolenic acid, arachidonic acid, cis-docosahexaenoic acid, gamma-linolenic acid, linolelaidic acid, linoleic acid, palmitoleic acid, cis-7-hexadecenoic acid, hexadecanoic acid, elaidic acid	Zuo et al. (2021) [30]
		123 DR 116 NDR	Metabolon DiscoveryHD4	Glycoursodeoxycholate, tryptophan, xanthine, phenylacetylglutamine, X-23997, X-13729, 1-palmitoyl-GPA (16:0), and 5-methylthioadenosine (MTA)	Yousri et al. (2022) [31]
	Erythrocyte	70 DR 14 controls	LC-MS	↓: arachidonic acid, docosahexaenoic acid, N-6 PUFAs, N-3 PUFAs	Koehrer et al. (2014) [32]
	Stool	45 PDR 90 NDR	UPLC-MS	Alantolactone, adenine, corosolic acid, desogestrel, D-erythro-sphinganine, HETE, leukotriene	Ye et al. (2021) [33]
		21 PDR 14 NDR	UPLC-MS	↑: betonicin, butylparaben, traumatic acid, thromboxane B3, salicyluric acid, pyro-L-glutaminyl-L-glutamine, harman, flazine, β-carboline ↓: D-proline, armillaramide, N-gamma-L-glutamyl-D-alanine, N-acetyl-L-methionine, L-threo-3-phenylserine, (R)-pelletierine	Zhou et al. (2021) [34]
	Retina	20 NPDR 20 NDR	UHPLC-MS	↓: long-chain ACs (C ≥ 14), longer-chain FAHFAs, DAGs, TAGs, PCs, Cer	Fort et al. (2021) [35]
	Aqueous humor	14 DR 13 NDR	NMR	↑: asparagine, DMA, glutamine, histidine, threonine ↓: lactate, succinate, 2HB	Jin et al. (2019) [36]
	Aqueous and vitreous humor	18 PDR 22 controls	LC-MS	Cysteine persulfides (CysSSH), cystine, oxidized glutathione trisulfide (GSSSG)	Kunikata et al. (2017) [37]
		Vitreous humor: 28 PDR 22 no diabetes Aqueous humor: 23 PDR 25 no diabetes	GC-MS	Vitreous humor: alanine, alloisoleucine, creatinine, glutamine, leucine, lysine, ornithine, pyroglutamic acid, pyruvic acid, phenylalanine, uric acid, threonine, valine, myoinositol, hydroxylamine; Aqueous humor: citrulline, D-glucose, isocitric acid, fructose 6-phosphate, L-lactic acid, threonic acid, myoinositol, D-2,3-dihydroxypropanoic acid	Wang et al. (2019) [38]
	Vitreous humor	2 PDR 2 PVR 7 no diabetes	NMR	unclear	Young et al. (2009) [39]
		22 PDR 22 no diabetes	NMR	↑: glucose, lactate ↓: ascorbic acid, galactitol	Barba et al. (2010) [40]
		16 NPDR 15 PDR 16 no diabetes	LC-MS	↑: 5-HETE ↓: 14(15)-EET, 11(12)-EET	Schwartzman et al. (2010) [41]
		20 PDR 31 no diabetes	HPLC-MS	↑: allantoin, arginine, citrulline, decanoylcarnitine, proline, ornithine, octanoylcarnitine, methionine	Paris et al. (2015) [42]
		9 PDR 8 controls	UHPLC-MS	Ascorbate, carnitine, citrulline, creatinine, dehydroascorbate, fumarate, glutamine, malate, N-amidino-L-aspartate, sn-glycerol 3-phosphate, proline, pyruvate, tripeptide, ribose, triacanthine, a-ketoglutarate, 5-oxoproline	Haines et al. (2018) [43]
		31 PDR 13 no diabetes	LC-MS	↑: 5-HETE, 12-HETE, 20-HETE, and 20-COOH-AA	Lin et al. (2020) [44]
		35 PDR19 no diabetes	UHPLC-MS	↑: allantoin, citrulline, dimethylglycine, glycine, lactate, ornithine, pyruvate, proline, urate, N-acetylserine, α-ketoglutarate ↓: creatine, succinate	Tomita et al. (2020) [45]
		41 PDR 22 no diabetes	UHPLC-MS	↑: 21 oxylipins (ARA, DHA, DTA, EPA, 8S-HETrE, 9-OxoODE, 9S-HOTrE, 9S-HODE, 13S-HOTrE, 13-OxoODE, ±12(13) -EpOME, 12S-HETE, ±12 (13)-DiHOME, ±9(10)-EpOME, ±9(10)-DiHOME, 13S(γ)-HOTrE, 15-deoxy-Δ12,14-PGJ2, 15S-HETrE, ±14,15-DiHETrE, ±19,20-EpDPE, and 13,14-dihydro PGF2α)	Zhao et al. (2022) [46]
	CSF and plasma	19 DR 14 controls	NMR	Alanine, histidine, leucine, pyruvate, tyrosine, and valine	Lin et al. (2019) [47]
	Plasma and serum	228 PDR 276 NPDR 141 NDR	GC-MS, UHPLC-MS	↑: 2,4-DHBA, 3,4-DHBA, 3,4-DHBA, ribitol ↓: LPC(16:1), PC(32:1), PC(32:2), TG(50:1), TG(50:2), TG(14:0/16:0/18:1), TG(50:3)	Curovic et al. (2020) [48]
	Plasma and vitreous humor	Plasma: 88 PDR 51 controlsVitreous: 51 PDR 23 controls	UPLC-MS	(↑ plasma and vitreous): pantetheine, (24R)-Cholest-5-ene-3-beta,24-diol, alpha-N-phenylacetyl-L-glutamine; (↓ plasma and vitreous): pipecolic acid; (plasma ↑, vitreous ↓): pyroglutamic acid	Wang et al. (2022) [49]
	Plasma, serum, and urine	666 DR 2211 NDR	NMR	Serum/plasma: cholesterol esters, creatinine, tyrosine Urine: citrate, ethanolamine, formate, hypoxanthine	Quek et al. (2021) [50]
**Rat**	Urine	6 DR rats 6 controls	UPLC-MS	↑: cholic acid, kynurenic acid, phenylacetylglycine, p-cresol sulfate, 3-methyldioxyindole, 5-l-glutamyl-taurine ↓: hippuric acid, indoxyl sulfate, p-cresol glucuronide	Wang et al. (2020) [51]
**Mice**	Plasma and retina	10 db/db mice 10 db/+ mice	LC-MS	133 lipids in plasma 61 lipids in retina 15 lipids in plasma and retina (e.g., DAG 34:2, DAG 38:5, LPC 18:1, PC 36:4, SM 36:2)	Sas et al. (2018) [52]
	Blood	20 db/db mice 10 db/m mice	UHPLC-MS	Arachidonic acid, cortisol, docosahexaenoic acid, lysoPC (18:0), leukotriene B4, prostaglandin D2, γ-linolenic acid	Ge et al. (2021) [53]
**Zebra-fish**	whole body	50 pdx1−/− zebrafish	UHPLC–MS	↑: glutamate, proline, taurine ↓: ornithine, spermidine, tyrosine	Wiggenhauser et al. (2021) [54]

DR, diabetic retinopathy; NDR, no diabetic retinopathy (with diabetes without diabetic retinopathy); PDR, proliferative diabetic retinopathy; NPDR, non-proliferative diabetic retinopathy; PVR, proliferative vitreoretinopathy; GC-MS, gas chromatography mass spectrometry; LC-MS, liquid chromatography mass spectrometry; HPLC-MS, high-performance liquid chromatography mass spectrometry; UPLC-LC, ultra-performance liquid chromatography mass spectrometry; UHPLC-MS, ultra-high-performance liquid chromatography mass spectrometry; NMR, nuclear magnetic resonance; CSF, cerebrospinal fluid.

**Table 2:** Prediction of potential biomarker of DR in human.

Samples	Cohorts	Biomarkers	AUC	Sensitivity	Specificity	Study
**Serum**	DR VS. NDR	A biomarker panel (12-HETE and 2-piperidone)	0.946	0.894	0.919	Xuan et al. (2020) [27]
	NPDR VS. NDR	A biomarker panel (12-HETE and 2-piperidone)	0.958	0.929	0.901	Xuan et al. (2020) [27]
	DR VS. NDR	A biomarker panel (linoleic acid, nicotinuric acid, ornithine, and phenylacetylglutamine)	0.920	0.960	0.780	Zuo et al. (2021) [30]
**Plasma**	DR VS. NDR	Cytidine	0.849	0.737	0.919	Xia et al. (2011) [17]
	DR VS. NDR	Adenosine	0.913	0.947	1.000	Xia et al. (2014) [18]
	DR VS. NDR	1,5-Gluconolactone, 2-deoxyribonic acid, gluconic acid, and urea	0.71, 0.68, 0.72, 0.69, respectively	unclear	unclear	Chen et al. (2016) [19]
	DR VS. NDR	Ratio of the levels of glutamine to glutamic acid	0.742	unclear	unclear	Rhee et al. (2018) [20]
	DR VS. NDR	A biomarker panel (alanine, histidine, leucine, pyruvate, tyrosine, and valine)	0.836	unclear	unclear	Lin et al. (2019) [47]
	PDR VS. NDR	Fumaric acid, uridine, acetic acid, and cytidine	0.96, 0.95, 1.00, 0.95, respectively	unclear	unclear	Zhu et al. (2019) [22]
	DR VS. NDR	A risk score (pseudouridine)	0.800	0.976	0.531	Sun et al. (2021) [24]
	PDR VS. (NPDR and NDR)	A risk score (pseudouridine, glutamate, leucylleucine and N-acetyltryptophan)	0.820	0.762	0.774	Sun et al. (2021) [24]
**Vitreous humor**	PDR VS. no diabetes	A biomarker panel (galactitol and ascorbic acid)	unclear	0.860	0.810	Barba et al. (2010) [40]
	PDR VS. no diabetes	Xanthine, proline, citrulline, pyruvate	1.000, 0.986, 0.972, 0.944, respectively	unclear	unclear	Haines et al. (2018) [43]
	PDR VS. no diabetes	DTA, EPA, DHA, ARA, ±9(10)-DiHOME, ±19,20-EpDPE, and ±12(13)-EpOME	0.960, 0.803, 0.871, 0.942, 0.805, 0.819, 0.828, respectively	unclear	unclear	Zhao et al. (2022) [46]
	PDR VS. no diabetes	A biomarker panel (pyroglutamic acid and pyruvic acid)	0.951	0.955	0.857	Wang et al. (2019) [38]
**Aqueous humor**	PDR VS. no diabetes	A biomarker panel (D-2,3-dihydroxypropanoic acid, isocitric acid, fructose 6-phosphate, and L-lactic acid)	0.965	0.880	0.957	Wang et al. (2019) [38]
**Cerebrospinal fluid**	DR VS. NDR	A biomarker panel (alanine, histidine, leucine, pyruvate, tyrosine, and valine)	0.858	unclear	unclear	Lin et al. (2019) [47]
**Stool**	PDR VS. NDR	A classifier (Top 5 are alantolactone, desogestrel, adenine, D-erythro-sphinganine, and corosolic acid.)	0.960	0.846	0.936	Ye et al. (2021) [33]

AUC, area under the ROC curve; HETE, hydroxyeicosatetraenoic acid; DTA, docosatetraenoic acid; EPA, eicosapentaenoic acid; DHA, docosahexaenoic acid; ARA, arachidonic acid; DiHOME, dihydroxy-octadecenoic acid; EpDPE, epoxy-docosapentaenoic acid; EpOME, epoxy-octadecenoic acid.

**Table 3:** Statistics of metabolic pathways associated with DR patients.

Samples	Pathways	Reported Times
**Plasma**	Purine metabolism	4
	Arginine and proline metabolism	3
	Pyrimidine metabolism	3
	Alanine, aspartate and glutamate metabolism	2
	Cysteine and methionine metabolism	2
	4-hydroxybenzeneacetic acid	1
	Arachidonic acid metabolism	1
	Aspartate and asparagine metabolism	1
	Caffeine metabolism	1
	Creatinine metabolism	1
	D-glutamine metabolism	1
	Fumaric acid metabolism	1
	Galactose metabolism	1
	Glyceryl-glycoside metabolism	1
	Histidine metabolism	1
	Leukotrienes metabolism	1
	Linoleic acid metabolism	1
	Lysine metabolism	1
	Myo-inositol metabolism	1
	Niacin metabolism	1
	Nitrogen metabolism	1
	Pantothenate and CoA biosynthesis	1
	Pentose phosphate metabolism	1
	Phenylalanine metabolism	1
	Polyol metabolism	1
	Riboflavin metabolism	1
	Sphingolipid metabolism	1
	Sulfur metabolism	1
	Urea cycle	1
	α-linolenic acid metabolism	1
**Vitreous**	Arginine and proline metabolism	2
	Valine, leucine, and isoleucine biosynthesis	2
	Alanine, aspartate and glutamate metabolism	1
	Aminoacyl-tRNA biosynthesis	1
	Glycine and serine metabolism	1
	Glycolysis	1
	Nitrogen metabolism	1
	Pantothenate and CoA biosynthesis	1
	Pentose phosphate pathway	1
	Phenylalanine metabolism	1
	Purine metabolism	1
	Taurine and hypotaurine metabolism	1
